# An Evaluation of the Physicochemical Properties of Preservative-Free 0.005% (*w*/*v*) Latanoprost Ophthalmic Solutions, and the Impact on In Vitro Human Conjunctival Goblet Cell Survival

**DOI:** 10.3390/jcm11113137

**Published:** 2022-05-31

**Authors:** Josefine C. Freiberg, Anne Hedengran, Steffen Heegaard, Goran Petrovski, Jette Jacobsen, Barbara Cvenkel, Miriam Kolko

**Affiliations:** 1 Department of Drug Design and Pharmacology, University of Copenhagen, 2100 Copenhagen, Denmark; josefine.freiberg@sund.ku.dk (J.C.F.); anne.hedengran.nagstrup@regionh.dk (A.H.); 2 Department of Ophthalmology, Copenhagen University Hospital, Rigshospitalet-Glostrup, 2600 Glostrup, Denmark; steffen.heegaard@regionh.dk; 3 Department of Pathology, Copenhagen University Hospital, Rigshospitalet, 2100 Copenhagen, Denmark; 4 Center for Eye Research, Department of Ophthalmology, Oslo University Hospital, 0450 Oslo, Norway; gokipepo@gmail.com; 5 Institute for Clinical Medicine, Faculty of Medicine, University of Oslo, 0372 Oslo, Norway; 6 Department of Pharmacy, University of Copenhagen, 2100 Copenhagen, Denmark; jette.jacobsen@sund.ku.dk; 7 Glaucoma Unit, Department of Ophthalmology, University Medical Centre Ljubljana, 1000 Ljubljana, Slovenia; barbara.cvenkel@gmail.com; 8 Medical Faculty, University of Ljubljana, 1000 Ljubljana, Slovenia

**Keywords:** latanoprost, preservative-free (PF), pH value, osmolality, surface tension, goblet cells, cell viability, tear film

## Abstract

Purpose: To examine the physicochemical properties of five preservative-free (PF) 0.005% latanoprost ophthalmic products; Monoprost^®^, Latanest^®^, Gaap Ofteno^®^, Xalmono^®^, and Xaloptic^®^ Free. Furthermore, the study investigated the mucin production and cell survival of primary cultured human conjunctival goblet cells when treated with PF eye drops. Method: The pH value, osmolality, and surface tension were examined. Cell survival was analyzed using lactate dehydrogenase and tetrazolium dye colorimetric assays. Mucin production was analyzed with immunohistochemical staining. Results: Monoprost^®^ (pH value 6.84 ± 0.032) had a pH value closest to the pH value of tear fluid (pH value 7.4–7.6), whereas Gaap Ofteno^®^ (pH value 6.34 ± 0.004) and Latanest^®^ (pH value 6.33 ± 0.003) had the lowest pH values. Gaap Ofteno^®^ (325.9 ± 2.9 mosmol/kg) showed iso-osmolar probabilities, whereas the other products were hypo-osmolar. Gaap Ofteno^®^ (60.31 ± 0.35 mN/m) had a higher surface tension compared to the tear fluid (40 to 46 mN/m), as described in the literature. No significant differences in goblet cell survival or mucin release were observed between the treatments and control. Conclusion: Significant differences in pH value, osmolality, and surface tension were observed. However, this did not affect the viability of the goblet cells or the release of mucin. Clinical studies are required to evaluate the long-term effects of use on efficacy and safety.

## 1. Introduction

Glaucoma is one of the leading causes of blindness worldwide, with an overall global prevalence of 3.54% (95%CI 2.09–5.82) in patients aged 40–80 years [1]. Elevated intraocular pressure (IOP) is a recognized risk factor in the development of glaucoma, and lifelong treatment is essential to prevent the progression of the disease [2,3,4]. Prostaglandin analog (PGA) eye drops are often the first choice when prescribing anti-glaucomatous eye drops because of their well-documented IOP-lowering effect and high tolerability [4].

Several studies have shown that long-term treatment with preservative-containing eye drops induces ocular surface inflammation, instability of the tear film, and damage to the corneal surface [5,6,7].

The tear film consists of a mucin/aqueous layer and an external lipid layer [8]. Mucin is produced by the conjunctival goblet cells. Goblet cells are essential for obtaining a stable tear film, as well as protecting and lubricating the ocular surface [8,9,10].

Various clinical, animal, and cellular studies document the finding that preservative agents, such as benzalkonium chloride (BAK), harm the ocular tissue and induce an inflammatory response [11,12,13,14].

Prostaglandins are lipophilic molecules and, thus, demand a solubilizing agent that is compatible with the tear film [15]. Besides the solution’s antibacterial properties, preservative agents such as BAK solubilize the ophthalmic solution. If preservative agents are removed from an ophthalmic solution, they should be replaced with other solubilizing or stabilizing agents. In 2012, a preservative-free (PF) 0.005% latanoprost ophthalmic solution with Macrogol glycerol hydroxy stearate 40 (MGH40) as a solubilizing agent was developed, patented, and marketed by Laboratoires Théa (Clermont-Ferrand, France) (Monoprost^®^) [16]. PF formulations can be dispensed as single-dose units or multidose bottles. To prevent contamination of the formulations with a water content above 20% (*w*/*w*), multidose containers have incorporated systems, such as filters or two-layer bottles, to protect against contamination. Pharmaceutical companies have patented multi-dose bottles, e.g., ABAK^®^ and EASYGRIP^®^, developed by Laboratoires Théa, Novelia^®^, developed by Nemerand (La Verpillière, France), and the 3K^®^ pump/COMOD^®^ device developed by Ursapharm (Saarbrücken, Germany) [17,18,19].

The European Medicines Agency (EMA, Amsterdam, The Netherlands) requires the active drug used in generics to be identical to the original preparation, with the same concentration, indication, route of administration, and bioavailability. However, the excipients may differ from the original preparation, and generics are not tested with the same efficacy and safety studies as the original products [20,21]. Differences in the physicochemical properties of preserved PGA eye drops have been identified [22,23], but little is known about the differences among PF PGA eye drops and their effect on the ocular surface, cells, and tissue.

In this pre-clinical study, five PF 0.005% latanoprost eye drops were investigated in terms of their chemical and physical properties: the pH value, osmolality, and surface tension. As a proxy for the eye drops’ effect on the viability of human conjunctival goblet cells, lactate dehydrogenase (LDH) and tetrazolium dye (MTT) colorimetric assays were conducted on cultured human conjunctival goblet cells. The presence of mucin in the goblet cells was evaluated with immunohistochemical staining.

## 2. Materials and Methods

### 2.1. Materials and Reagents

We used the same methods as previously described by Müllerts et al. and Hendegran et al. [24,25]. The following 0.005% (*w*/*v*) PF latanoprost products were included: Monoprost^®^ (Laboratoires Théa—France), Latanest^®^ (Esteve—Spain), Gaap Ofteno^®^ (Laboratorios SOPHIA—Mexico), Xalmono^®^ (Rockmed—Belgium) and Xaloptic^®^ Free (Polpharma, Poland)

In brief, human goblet cells from donors were cultured in Roswell Park Memorial Institute (RPMI) media 1640 1x (32404-014; Gibco, Life Technologies, Waltham, MA, USA) with 10% fetal bovine serum (FBS) (10270-106; Gibco, Life Technologies, Waltham, MA, USA) and 1% of the following mentioned solutions: penicillin/streptomycin (15140-122; Gibco, Life Technologies, Waltham, MA, USA), non-essential amino acid (NEAA) solution (M7145; Sigma-Aldrich, St. Louis, MO, USA), 1 M HEPES (15630-080; Gibco, Life Technologies, Waltham, MA, USA), L-glutamine (25030-024; Gibco, Life Technologies, Waltham, MA, USA) and sodium pyruvate (11360-039; Gibco, Life Technologies, Waltham, MA, USA).

When performing LDH and MTT assays, phosphate-buffered saline (PBS) was prepared with 137 mM NaCl, 2.7 mM KCl, 10 mM Na_2_HPO_4_, and 1.8 mM KH_2_PO_4_ with a pH value of 7.4, adjusted with either 1 M HCl or 1 M NaOH. Then, 1 M EDTA (E5134; Sigma-Aldrich, St. Louis, MO, USA) in PBS, 0.48 mM versene (15040-033; Gibco, Life Technologies, Waltham, MA, USA), and trypsin (T4799; Sigma-Aldrich, St. Louis, MO, USA) were used when trypsinizing the goblet cells. An LDH cytotoxicity detection kit from Takara BIO, Kusatsu Shiga, Japan (MK401) was utilized, and the MTT assay was performed using 12.5 mM thiazolyl blue tetrazolium bromide (M5655; Sigma-Aldrich, St. Louis, MO, USA) in PBS.

For immunohistochemical staining, 4% (*w*/*v*) of paraformaldehyde in PBS (provided by the RegionH pharmacy) was used to fixate the cells. PBS, Triton-X (1001325622; Sigma-Aldrich, St. Louis, MO, USA), bovine serum albumin (ab181831; Sigma-Aldrich, St. Louis, MO, USA), and saponin from Quillaja Bark (1001658552; Sigma-Aldrich, St. Louis, MO, USA) were used for immunohistochemical staining with the primary antibodies, Cytokeratin7 (ab181831; Abcam, Cambridge, UK) and monoclonal anti-human gastric mucin (M5293; Sigma-Aldrich, St. Louis, MO, USA). The secondary antibodies used were Alexa488 (A11034; Gibco, Life Technologies, Waltham, MA, USA), Texas red (T862; Gibco, Life Technologies, Waltham, MA, USA), and DAPI (D3571; Invitrogen, Waltham, MA, USA).

### 2.2. Physicochemical Characterization

The osmolality and pH values were measured in triplicate for both diluted and undiluted eye drops. The eye drops were diluted at 1:7 (*v*/*v*) in RPMI media. The pH value was measured at room temperature using a calibrated 744 pH meter (Metrohm; Nordic ApS, Herisau, Switzerland), and the freezing point depression (Osmomat 3000; Gonotec, Berlin, Germany) measured the osmolality. The Wilhelmy method was used to detect the surface tension with a force tensiometer, K-100c (Krüss GmbH, Hamburg, Germany), and the Laboratory Desktop software, version 3.2.2.3068 (Laboratory Desktop, Krüss GmbH). The measurements were performed in triplicate with the undiluted products at room temperature and with a standard deviation of less than 0.1 mN/m.

### 2.3. Human Conjunctival Goblet Cell Cultivation

With approval from the Danish National Committee on Health Research (H-17007902) and the Norwegian Regional Committees for Medical and Health Research Ethics (REK: 2013/803), the conjunctiva from post-mortem human donors was cultivated to generate primary goblet cell cultures. Conjunctiva pieces were incubated for 14 days at 37 °C and 5% CO_2_. Media was added to the cultures every day for the first three days and, thereafter, was changed every other day. To keep the purified cell cultures, microscopy of the cells was performed before every media change using light microscopy. If any fibroblasts appeared in the cultures, they were removed manually.

### 2.4. Cell Survival Analysis

The goblet cells were trypsinized after 14 days of cultivation, counted, and replated in a 96-well plate with a cell density of 25,000 cells/cm^2^ for LDH and 50,000 cells/cm^2^ for the MTT assay. Cells were incubated for an additional four to five days at 37 °C and 5% CO_2,_ to ensure adhesion before they were treated for 30 min with eye drops that were diluted at 1:7 (*v*/*v*) in media. After 30 min of treatment, the eye drops were removed, fresh media was added, and the cells were incubated for various time periods, depending on the assay performed thereafter. The LDH assay was performed 20 h after treatment with eye drops. LDH solution was prepared immediately before the experiment, added to the cells, and incubated at room temperature in the dark for 15–20 min before the stop solution (10% HCL, *v*/*v*) was added. Concerning the MTT assay, 12 mM MTT (*w*/*v*) in PBS was added to the cells, immediately after treatment with the diluted eye drops. The cells were incubated at 37 °C and 5% CO_2_ for one hour, followed by adding 0.01% (*v*/*v*) HCL in 10% (*w*/*v*) SDS, in PBS solution. The cells were then incubated in the dark for 18 h at room temperature. A SpectraMax i3X multi-mode microplate reader (Molecular Devices, San Jose, CA, USA) with an absorbance of 490 nm for LDH and 560 nm for MTT was applied. To secure the reproducibility of the results, a minimum of three batches from a minimum of four different donors was required for analysis. For every experiment, a control treated with only RPMI media was included. The cell survival data was calculated as the mean percentage change in absorbance, compared to the control ± standard deviation (SD).

### 2.5. Immunohistochemical Staining

The goblets cells were cultivated on slides and treated with RPMI media or diluted eye drops 1:7 (*v*/*v*) for 30 min at 37 °C 5% CO. With the use of paraformaldehyde 4% (*v*/*v*), the slides were fixated and stored at 4 °C. The cell membranes of the goblet cells were permeated using 0.1% *v*/*v* Triton X-100 in PBS, and by using 3% (*w*/*v*) bovine serum albumin in PBS, unspecific binding was blocked. The cells were treated with the primary antibodies Cytokeratin-7 (anti-cytokeratin7, 1:500 *v*/*v*) and monoclonal anti-human gastric mucin (anti-mucin, 1:200 *v*/*v*), diluted in 0.25% bovine serum albumin/0.1% saponin in PBS and washed with PBS thereafter. The fluorescent secondary antibodies, Alexa488 (anti-rabbit, 1:500 *v*/*v*) and Texas red (anti-mouse, 1:200 *v*/*v*), both diluted in 0.25% bovine serum albumin/0.1% saponin in PBS, were added. Then, 0.3 µM of DAPI in PBS stained the nuclei of the goblet cells. Imaging was performed using an Axioskop 2 (Zeiss; Göttingen, Germany) with an Axio Cam MRm camera (Zeiss; Göttingen, Germany) and an HXP 120 lighting unit (Zeiss; Göttingen, Germany). Image scaling and the merging of pictures were conducted using ImageJ 1.52q (Wayne Rasband, National Institute of Health, Bethesda, MD, USA).

### 2.6. Statistics

The software program, GraphPad Prism 9 (GraphPad Software, San Diego, California USA), was used for the statistical analyses and graphs. Descriptive statistics and a comparative one-way analysis of variance (ANOVA) were chosen as the statistical analyses for all data sets. To estimate the differences in cell survival, osmolality, pH value, and surface tension among the treatments, Tukey’s multiple comparison test was applied. All results were expressed as mean ± SD, and a *p*-value of ≤ 0.05 was considered statistically significant.

## 3. Results

### 3.1. pH Value

**Figure 1:**
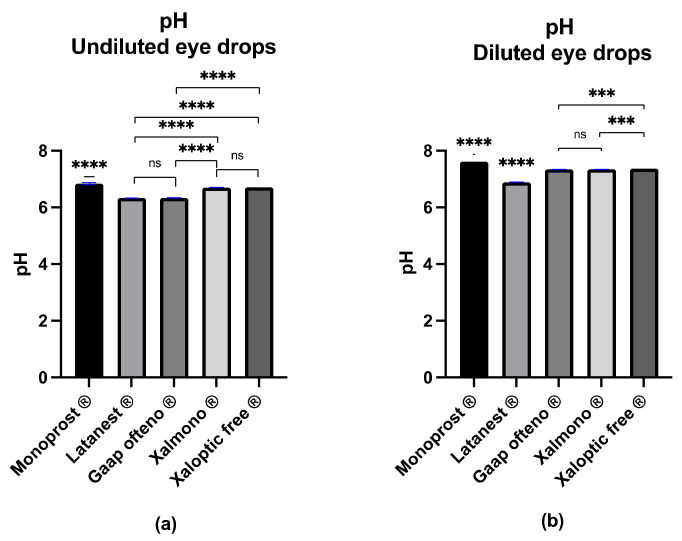
The pH value characterization of preservative-free 0.005% latanoprost products Monoprost^®^, Latanest^®^, Gaap Ofteno^®^, Xalmono^®^, and Xaloptic^®^ Free: (**a**) pH value of the undiluted eye drops; (**b**) pH value of the diluted eye drops (1:7, *v*/*v*). Values are listed as mean ± SD, and *n* = 3. A one-way ANOVA with a Tukey multiple-comparison test (*p* = 0.05) was performed. ns = not significant, with *p* ≥ 0.05, *** *p* ≤ 0.001, **** *p* ≤ 0.0001.

The pH value was examined in both undiluted and diluted products, while the goblet cell cultures were treated with diluted products (Figure 1). Of the undiluted eye drops (Figure 1a), Monoprost^®^ had the highest pH value of 6.84 ± 0.032, while Latanest^®^ had the lowest pH value of 6.33 ± 0.003. The pH value of the remaining eye drops was 6.34 ± 0.004 (Gaap Ofteno^®^), 6.70 ± 0.003 (Xalmono^®^), and 6.71 ± 0.000 (Xaloptic^®^ Free). Significant differences (*p* ≤ 0.0001) were observed between all eye drops, except between Latanest^®^ and Gaap Ofteno^®^, and between Xalmono^®^ and Xaloptic^®^ Free. As presented in Figure 1b, the pH value of the diluted formulations was 7.62 ± 0.002 (Monoprost^®^), 6.89 ± 0.007 (Latanest^®^), 7.34 ± 0.004 (Gaap Ofteno^®^), 7.34 ± 0.009 (Xalmono^®^) and 7.37 ± 0.002 (Xaloptic^®^ Free). Significant differences were observed between the eye drops with *p* ≤ 0.001 or *p* ≤ 0.0001. Gaap Ofteno^®^ and Xalmono^®^ were not significantly different.

### 3.2. Osmolality

**Figure 2:**
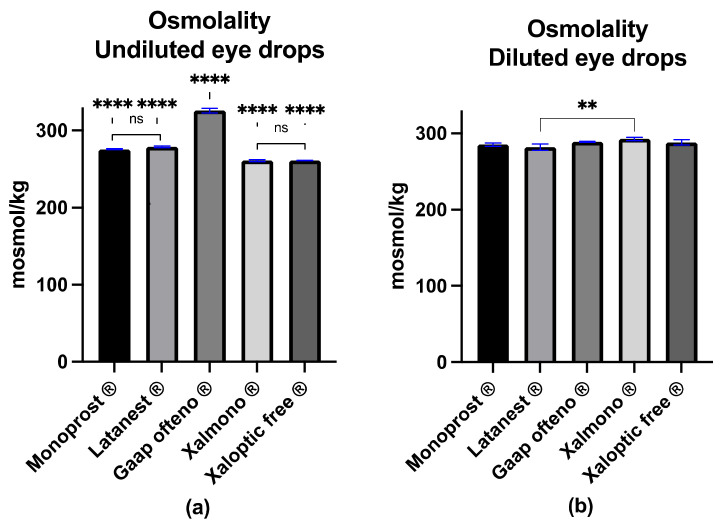
Osmolality characterization of preservative-free 0.005% latanoprost eye drops Monoprost^®^, Latanest^®^, Gaap Ofteno^®^, Xalmono^®^ and Xaloptic^®^ Free: (**a**) osmolality of the undiluted eye drops; (**b**) osmolality of the diluted eye drops (1:7, *v*/*v*). Values are listed as mean ± SD, and *n* = 3. A one-way ANOVA with a Tukey multiple-comparison test (*p* = 0.05) was performed. ns = not significant with *p* ≥ 0.05, ** *p* ≤ 0.01, **** *p* ≤ 0.0001.

The osmolality was examined in both undiluted and diluted products, while the goblet cell cultures were treated with diluted products (Figure 2). The osmolality of the undiluted Gaap Ofteno^®^ was 325.9 ± 2.9 mosmol/kg and was significantly higher, compared to the four other products (*p* ≤ 0.0001) (Figure 2a). The osmolality of the other eye drops was 275.8 ± 0.7 mosmol/kg (Monoprost^®^), 278.9 ± 1.3 mosmol/kg (Latanest^®^), 261.0 ± 1.2 mosmol/kg (Xalmono^®^) and 261.2 ± 0.3 mosmol/kg (Xaloptic^®^ Free), as visualized in Figure 2a. There were no significant differences between Monoprost^®^ and Latanest^®^ or between Xalmono^®^ and Xaloptic Free^®^.

Of the diluted eye drops (Figure 2b), Latanest^®^ had the lowest osmolality of 281.9 ± 4.2 mosmol/kg, and Xalmono^®^ had the highest osmolality of 292.7 ± 2.1 mosmol/kg. Monoprost^®^ had an osmolality of 285.3 ± 2.2 mosmol/kg, Xaloptic^®^ Free, osmolality of 288.1 ± 3.5 mosmol/kg, and Gaap Ofteno^®^, osmolality of 288.9 ± 0.5 mosmol/kg (Figure 2b). Significant differences (*p* ≤ 0.01) were observed between Latanest^®^ and Xalmono^®^.

No significant differences were observed among the other eye drops.

### 3.3. Surface Tension

**Figure 3:**
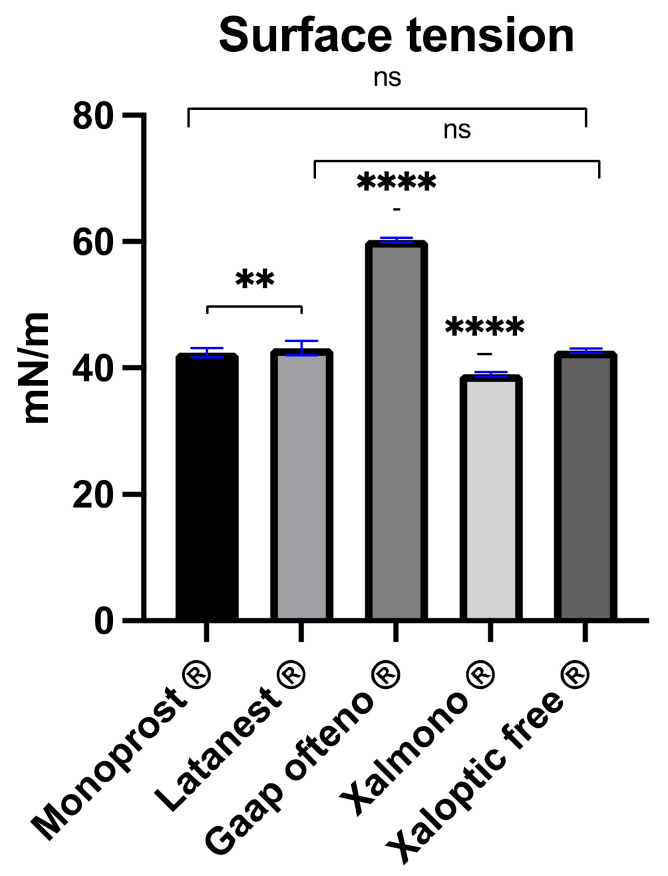
Surface tension characterization of preservative-free 0.005% latanoprost products; Monoprost^®^, Latanest^®^, Gaap Ofteno^®^, Xalmono^®^ and Xaloptic^®^ Free. Values are listed as mean ± SD, *n* = 3. A one-way ANOVA with a Tukey multiple-comparison test (*p* = 0.05) were performed. ns = not significant with *p* ≥ 0.05, ** *p* ≤ 0.01, **** *p* ≤ 0.0001.

Gaap Ofteno^®^ demonstrated a surface tension of 60.31 ± 0.35 mN/m, which was significantly higher compared to the other products (*p* ≤ 0.0001) (Figure 3). Xalmono^®^ had the lowest surface tension of 39.00 ± 0.38 mN/m, and was significantly lower compared to the other products (*p* ≤ 0.0001). No significant differences were observed between Monoprost^®^ (42.44 ± 0.75 mN/m) and Xaloptic^®^ Free (42.76 ± 0.36 mN/m), or between Latanest^®^ (43.15 ± 1.13 mN/m) and Xaloptic^®^ Free.

### 3.4. Cell Survival

**Figure 4:**
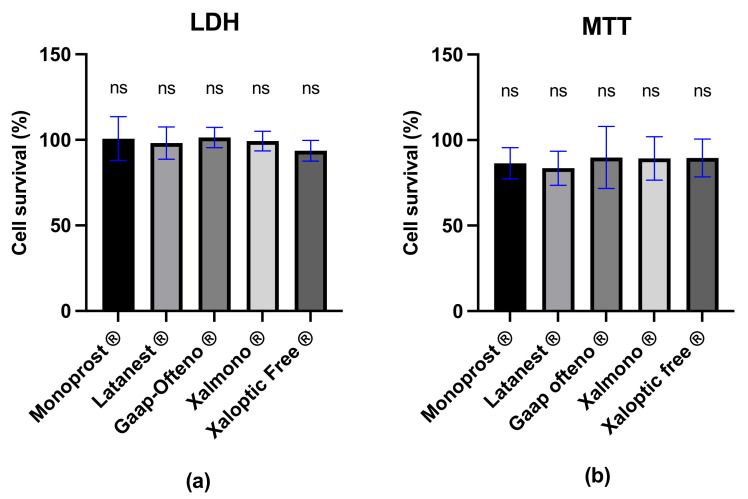
Mean cell survival analysis of human conjunctival goblet cells in % ± SD, relative to control, after 30 min of treatment with preservative-free 0.005% latanoprost eye drops. (**a**) Cell survival, examined with the LDH assay; (**b**) cell survival, examined with the MTT assay. Goblet cells were treated with diluted (1:7, *v*/*v*) preservative-free 0.005% latanoprost products: Monoprost^®^, Latanest^®^, Gaap Ofteno^®^, Xalmono^®^, and Xaloptic^®^ Free. A one-way ANOVA with a Tukey multiple-comparison test was performed (*p* = 0.05), *n* ≥ 4; ns = not significant; *p* ≥ 0.05.

LDH and MTT analyses were performed on primary cultured human conjunctival goblet cells treated with diluted PF 0.005% latanoprost products. Cell survival was reported relative to the control, these being goblet cells treated with RPMI media. According to the results of the LDH assays, the mean cell survival after treatment with eye drops was 100.70 ± 12.8% (Monoprost^®^), 98.17 ± 9.4% (Latanest^®^), 101.40 ± 5.9% (Gaap Ofteno^®^), 99.36 ± 5.7% (Xalmono^®^) and 93.66 ± 6.0% (Xaloptic^®^ Free). As seen in Figure 4a, the LDH assay showed no significant differences in cell survival between treatments. Similar results were obtained from the MTT analysis, showing no significant differences in cell survival between the treatments (Figure 4b). The mean cell survival compared to the control was 86.40 ± 9.1% (Monoprost^®^), 83.53 ± 9.9% (Latanest^®^*)*, 89.84 ± 18.2% (Gaap Ofteno^®^), 89.30 ± 12.7% (Xalmono^®^), and 89.51 ± 11.0% (Xaloptic^®^ Free).

### 3.5. Immunohistochemical Staining

**Figure 5:**
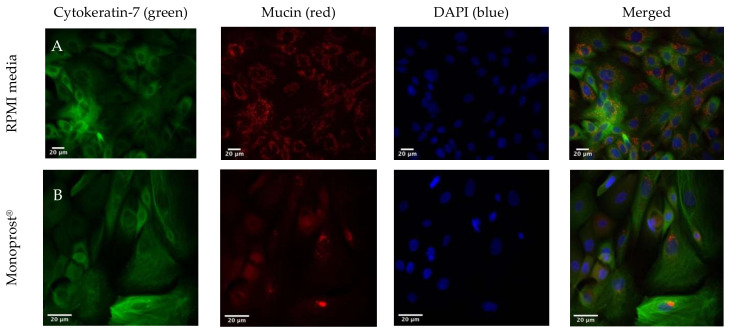

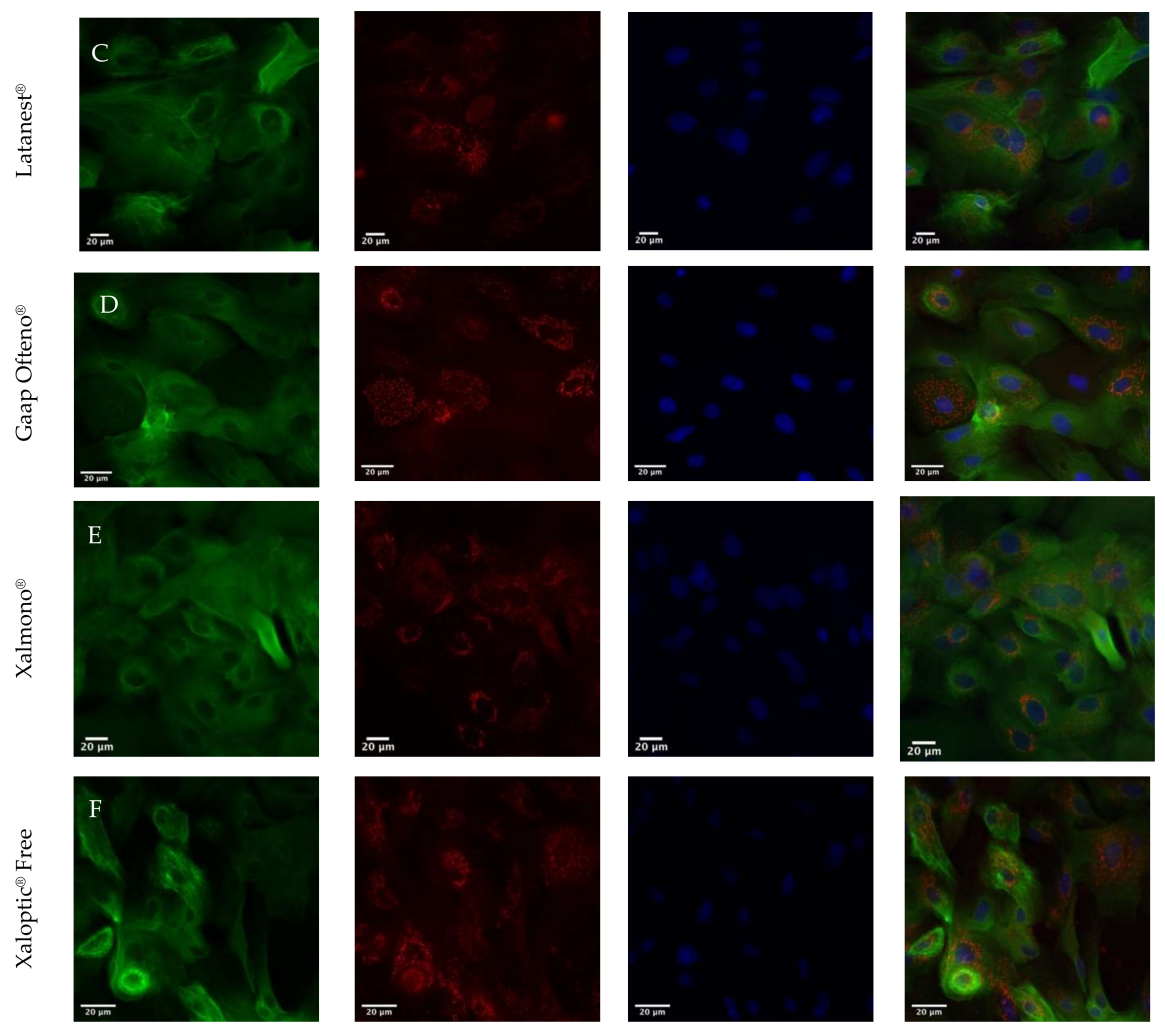
Immunohistochemical staining of human goblet cells, visualizing the cytoskeleton (column 1: Cytokeratin-7, green), mucin (column 2: mucin, red), the nucleus (column 3: DAPI, blue), and merged stainings (column 4: cytokeratin-7 (green), mucin (red) and DAPI (blue)). Cells were treated with diluted (1:7, *v*/*v*) 0.005% latanoprost preservative-free products. (**A**): RPMI media, (**B**): Monoprost ^®^, (**C**): Latanest^®^, (**D**): Gaap Ofteno^®^, (**E**): Xalmono^®^, and (**F**): Xaloptic^®^ Free. *n* = 3.

Immunohistochemical staining was used to visualize the cytoskeleton of the goblet cells, the nuclei, and mucin, as seen in Figure 5. Goblet cells treated with RPMI media showed mucin allocated around the nuclei (Figure 5A). Figure 5B–F demonstrated a similar pattern, showing mucin allocated near the nuclei when the cell cultures were treated with diluted PF 0.005% latanoprost eye drops: Monoprost^®^, Latanest^®^, Gaap Ofteno^®^, Xalmono^®^, and Xaloptic^®^ Free.

## 4. Discussion

Anti-glaucomatous treatment is lifelong, and the importance of minimizing adverse effects is crucial to increasing patients’ adherence to the treatment regimen and for health-related quality of life [26]. Glaucoma patients experience side effects from the treatment, such as dry eye disease (DED), a foreign-body sensation, and a stinging or burning sensation in the eye [27]. The cause of DED is either reduced lacrimation or increased evaporation from the ocular surface. Changes in the ocular surface may lead to a hyperosmolar condition, which is a recognized risk factor for inducing proinflammatory stress [8,28,29]. The instability of the tear film can cause ocular discomfort or irritation, while pH value, osmolality, and surface tension all play a role in achieving a healthy ocular surface. The current study revealed significant differences in the physicochemical properties of pH value, osmolality, and surface tension. Some of the variations may be due to the product formulation, as Monoprost^®^, Xaloptic^®^ Free, and Xalmono^®^ were dispensed from single-dose units, whereas Gaap Ofteno^®^ and Latanest^®^ were dispensed from multi-dose bottles. Osmolality, pH value, and surface tension may vary due to variations in the solvents or stabilizers caused by the removal of preservatives. The physicochemical properties of the undiluted eye drops illustrated the properties of the eye drops immediately after application to the ocular surface in patients.

The pH value of tear fluid is 7.4–7.6 [30,31,32]. To optimize ocular comfort, the pH value of the ophthalmic formulations should match the tear film or at least be in the ocular range of 6.6–7.8, as an acidic or alkaline pH value induces lacrimation, ocular pain, and discomfort [23,32]. In this study, we found that Latanest^®^ (pH value 6.33 ± 0.003) and Gaap Ofteno^®^ (pH value 6.34 ± 0.004) were acidic, with pH values below the ocular range. The remaining three products, Monoprost^®^ (pH value 6.84 ± 0.032), Xalmono^®^ (6.70 ± 0.003), and Xaloptic^®^ Free (6.71 ± 0.000), had pH values within the ocular range, with Monoprost^®^ closest to the pH value of the tear film. An acidic pH value may cause side effects, such as ocular discomfort and increased lacrimation upon instillation, whereas a pH within the recommended range should not provide any discomfort related to the pH value. When the products were diluted with RPMI media, the pH values of all products were within the recommended pH range. Our findings imply that it would be of great interest to investigate the pH value of PF PGA products upon dilution with tear fluid in patients, to elucidate if differences in pH value may cause prolonged ocular discomfort.

The osmolality of the tear fluid varies from 310 to 350 mosmol/kg [31]. All products except Gaap Ofteno^®^ (325.9 ± 2.9 mosmol/kg) were hypo-osmolar, compared to the tear fluid. As previously mentioned, a hyperosmolar tear film is related to ocular irritation and DED. Based on our findings, the osmolality of the tested products should not be of particular concern, as they demonstrated iso- or hypo-osmolar properties.

The surface tension of the tear fluid varies from 40 to 46 mN/m and ensures a stable tear film and tear film break-up time [33,34]. Furthermore, the surface tension influences the eye drops’ ability to spread and adhere to the cornea, once applied [34]. We found that Monoprost^®^ (42.44 ± 0.75 mN/m), Xaloptic^®^ Free (42.76 ± 0.36 mN/m), and Latanest^®^ (43.15 ± 1.13 mN/m) had a surface tension in the physiological range of the tear fluid. Gaap Ofteno^®^ (60.31 ± 0.35 mN/m) had a surface tension well above the physiological range, while Xalmono^®^ had a surface tension just below 40 (39.0 ± 0.38 mN/m). Surface tension exceeding the physiological range may cause instability of the tear film and is associated with dry eyes [34]. In addition, higher surface tension will increase the drop volume released from the bottle [35]. The drop volume will also affect the amount of latanoprost released [23]. A greater drop volume will increase the washout and may result in less uptake of latanoprost and reduced efficacy in terms of lowering IOP. Thus, increased surface tension in ophthalmic solutions may lead to both the ocular adverse effects caused by a destabilized tear film and potentially reduce the efficacy of the eye drops.

Keeping goblet cells unharmed and unstressed is essential for maintaining a stable tear film for a healthy ocular surface [9,10]. Diluted PF 0.005% latanoprost eye drops showed no negative effects on either cell survival or mucin release after treatment, according to the LDH assay, MTT assay, and immunohistochemical staining. Other studies have investigated the differences between preserved and preservative-free PGAs. Treatment with PF tafluprost showed reduced pro-apoptotic and pro-oxidative stress in a conjunctival epithelial cell line, compared to preserved PGAs [36]. In patients, the goblet cell density was significantly increased after six months of treatment with PF tafluprost, compared to the baseline. In comparison, patients treated with preserved tafluprost showed increased goblet cell density after one month of treatment, but no significant long-term effects were reported [37].

As previously mentioned, it is of great interest to minimize the adverse effects of treatment, since glaucoma is a chronic condition. A frequently reported side effect of PGAs is conjunctival hyperemia [38,39]. PF PGAs, e.g., Monoprost^®^ and Gaap Ofteno^®^, have previously been examined and compared to preserved PGAs. A meta-analysis of 21 studies found that conjunctival hyperemia was significantly reduced in Monoprost^®^ compared to preserved PGAs, with no significant differences in IOP between treatments [40]. In addition, Rouland et al. showed noninferiority in IOP efficacy and a significantly reduced conjunctival hyperemia after treatment with Monoprost^®^, compared to treatment with Xalatan^®^ [41].

Gonzales et al. compared the stability, efficacy, and adverse effects of Gaap Ofteno^®^ with the preserved brand product, Xalatan^®^ [42]. As Gaap Ofteno^®^ is dispensed from a multidose bottle, the stability of the drug was also examined. The study showed that the two products were comparable in terms of IOP reduction and safety, evaluated as conjunctival hyperemia, with a similar prevalence of 11.3% (Gaap Ofteno^®^) and 11.9% (Xalatan^®^). The above-mentioned studies support our findings that PF 0.005% latanoprost products are non-toxic to human conjunctival goblet cells under experimental conditions.

When patients instill ophthalmic solutions in the eye, the tear film dilutes the ophthalmic solutions and only 1–7% of the instilled drug reaches the aqueous humor [43]. We chose to treat goblet cell cultures with diluted eye drops at a constant concentration for 30 min. In patients, the tear film dilutes the eye drops gradually upon administration, with a reduced expected exposure time (less than 30 min) as the fluid turnover rate of the conjunctival cul-de-sac in the eye is 0.5–2.2 μL/min. With this turnover rate, the drug remains in the conjunctival cul-de-sac for approximately 3–5 min [43]. The RPMI media used in this study was not identical to the tear film and will have different capacities, for instance, in buffering. Therefore, the physicochemical properties of eye drops diluted with tear film may affect the goblet cells in other ways than those identified in this study.

The in vitro model used in this study has some limitations since it cannot be directly compared to the conditions in patients. We did not quantify the amount of mucin released from goblet cells; the immunohistochemical staining that was performed only detected the presence of mucin. In addition, we did not evaluate the long-term effects of treating human conjunctival goblet cells with PF products. Thus, clinical trials would be desirable to examine whether the physicochemical differences addressed in this study may influence the long-term efficacy and safety profile of PF eye drops.

## 5. Conclusions

In conclusion, this study identified significant differences in pH value, osmolality, and surface tension among five PF 0.005% latanoprost products. The variations in physicochemical properties, such as acidic pH values or high surface tension, may potentially destabilize the tear film and reduce the tolerability of eye drops on the ocular surface. However, the variations in the physicochemical properties of the PF eye drops had no negative effects on either cell survival or mucin release. Since the efficacy was not examined in this in vitro experiment, clinical studies would be of great interest in elucidating the potential differences among PF PGA treatments, in terms of efficacy, tolerability, and the side effects related to long-term treatment with PF 0.005% latanoprost products.

## Data Availability

Datasets are available on reasonable request.
